# Spectrally-resolved errors in absorption and reduced scattering due to *µ*_a_-*µ*_s_’ cross-talk in layered media

**DOI:** 10.1364/BOE.573069

**Published:** 2025-11-13

**Authors:** Zachary D. Jones, Dominik Reitzle, Alwin Kienle

**Affiliations:** 1 Das Institut für Lasertechnologien in der Medizin und Messtechnik an der Universität Ulm (ILM), Germany; 2 Department of Physics, Universität Ulm, Germany

## Abstract

Spatially-resolved reflectance (SRR) techniques are essential for *in vivo* noninvasive optical characterization of biological tissue. Under the common assumption of a single-layer volume to approximate human skin tissue, the long-standing issue of *µ_a_*-*µ_s_*^’^ cross-talk warrants a thorough investigation and description of its potentially detrimental effects on the accuracy of measured absorption and reduced scatter. Using a two-layer model built from *ex vivo* measured porcine optical properties, we use analytical solutions to the radiative transfer equation to obtain calculated reflectance curves, which are fitted with a single-layer model to determine effective optical properties. We demonstrate systematic errors in fitted optical coefficients that display clear dependence on the optical properties of the two-layer medium and the inversion cost function. We provide a guide of the errors that a researcher may expect when performing *in vivo* optical characterization of biological tissue with SRR methods under the single-layer inverse model.

## 1. Introduction

### 1.1. Overview of spatially-resolved reflectance (SRR)

Reflected light from a material carries with it valuable information, and many techniques have been developed to leverage this for optical characterization and biomedical sensing applications [1–7]. Among these are spatially-resolved reflectance (SRR) measurement techniques in which systems typically consist of a single light source paired with several spatially-resolved detectors [8,9]. Often, a collection of fibers is used, one for incident light delivery to the sample and a number of others positioned at unique source-detector separations (SDS) to collect light that has propagated through and reemitted from the sample. Imaging SRR modalities also exist in which a camera is used to obtain an image of the illuminated sample, with spatial binning applied to images to retrieve a characteristic SRR curve [10,11]. The radiative transport theory and corresponding radiative transfer equation (RTE) serve as a standard theoretical model for light propagation in absorbing turbid media [12]. Under certain geometric conditions, one can obtain analytical solutions to the RTE [13] with which one can generate reference SRR curves. In media in which the RTE cannot be solved analytically, one may acquire numerical solutions with Monte Carlo (MC) simulations using the unique sample and detector geometry [14]. Whether solved analytically or numerically, RTE solutions provide reference SRR curves that can be compared to experimental ones, thus allowing for measurements of the absorption coefficient *µ_a_* and reduced scattering coefficient *µ_s_*^’^ by minimization of a cost function (by inversion methods such as a Levenberg-Marquardt (LM) algorithm), by matching to a precalculated look-up table of Monte Carlo (MC) simulated SRR curves, or by neural network (NN).

The coefficients *µ_a_* and *µ_s_*^’^ efficiently describe the optical characteristics of turbid media, allowing SRR methods to find use in a number of fields, including agricultural and food applications, in which the optical properties of fruits have been seen to change in response to diseased states [15], and geological settings in measurements of the optical properties of sea ice – an indicator of environmental health [16]. The most common application of SRR techniques is for the optical characterization of superficial human tissues, including the epidermis, dermis (and its sublayers such as papillary and reticular dermis), subcutaneous tissue, and muscle [8,17–23]. Because SRR techniques are reliant only on reflected and not transmitted light, they are uniquely suited for *in vivo* measurements of such tissues, as opposed to integrating sphere techniques which most often require excision, and thus are a good choice for *ex vivo* measurements of human tissue optical coefficients. Published human tissue optical properties are applied in a variety of ways, with a popular application being the modeling of photoplethysmography (PPG) by many-layered MC simulations for investigations in pulse oximetry or other vital sign monitoring [24–30]. Accurate *in vivo* skin and other human tissue optical properties are also necessary for physically-realistic rendering applications in which layered light propagation models accurately reproduce the appearance of human skin [31]. Thus, for applications in which an accurate optical model of human skin is to be built, accurate values of relevant tissue optical properties are necessary.

### 1.2. Choice of reference model

In experimental measurements of relevant human tissue optical properties, one may choose either a single- or multi-layered model from which to obtain reference SRR data for optimization and fitting of *µ_a_* and *µ_s_*^’^. While a semi-infinite model is defined optically by only one set of optical parameters: *µ_a_*, *µ_s_*^’^, refractive index *n*, and anisotropy of scatter *g*, a multilayer model possesses a unique set of optical parameters for all layers and a thickness *l* of each finite layer. Historically, during *in vivo* optical characterization of skin by SRR methods, the tissue volume has most often been approximated to be geometrically and optically homogenous, despite it being well-known that skin is highly layered and sub-layered [32,33], with non-uniform volume distributions of chromophores such as melanin and hemoglobin. The assumption of a single-layer volume in obtaining one’s reference SRR curves for comparison to experiment raises the important question of whether the fitted effective *µ_a_* and *µ_s_*^’^ can accurately represent the somehow averaged optical properties of each individual layer. It has been long-known that, when making the assumption of an optically homogenous skin medium, a phenomenon of *µ_a_*-*µ_s_*^’^ cross-talk can arise in the inversely fitted optical properties [1,10,34,35], in which a change in a single optical coefficient results in a response in the inversely fitted result of a coefficient that did not experience a perturbation during the measurement. These issues are still very important today, with relevant work ongoing to consider the errors that may arise during physiological measurements [36,37].

In a study with SRR simulated data, Farrell et al. constructed a two-layer model, with each layer having distinct *µ_a_* and *µ_s_*^’^, and forward reflectance curves were obtained while varying only one of four total coefficients. They observed that, when the two-layer forward curves were fitted by a two-parameter LM inverse algorithm built upon a single-layer reference model, fittings resulted in the repeatable “cross-talk” of a given optical coefficient into the fitted value of another [34]. Further, their results demonstrated the dependence of cross-talk on SDS and on the used inverse fitting algorithm. Hennessey et al. constructed a two-layer model from which forward SRR curves were obtained while holding all coefficients but one constant and subsequently fitting with both a single- and two-layer reference model [38]. They determined that the fitted melanin concentration was underestimated when fitting with a single-layer reference model, with the degree of error dependent on the top layer thickness. Recently, in an integrating sphere study of layered phantoms and leaves, which possess optically-distinct layers, Konrad et al. observed significant over- or underestimation of fitted optical properties in response to sample orientation when using a single-layer reference model assumption [39]. In a recent work, Jones et al. built a two-layer model to obtain forward reflectance using an analytical solution to the RTE under the constraint of equal absorption in both layers [40]. Fittings of *µ_a_* and *µ_s_*^’^ revealed that systematic under- and overestimation of absorption and chromophore concentrations may occur when using a single-layer to model a two-layer medium.

Alternatively, there has been much work in recent years to improve inverse fitting approaches for the accurate retrieval of optical coefficients using a multi-layered reference model. It has been demonstrated that, with both optimization algorithms and NNs, it is difficult to obtain accurate fits for all five parameters of top and bottom layer *µ_a_* and *µ_s_*^’^ and *l* in a two-layer model without significant *a priori* information. Tseng et al. obtained simulated reflectance curves arising from a two-layer medium and performed fittings with an LM inverse algorithm [41]. The values of the five fitting parameters of a two-layer model were recovered, with their accuracy typically improving in the case of known *l*. In a similar approach from Cen and Lu using hyperspectral SRR, the four optical coefficients in a two-layer model were fitted from simulated reflectance curves and were found to possess relatively low errors from their true values under the condition that *l* was known [42]. In a spatially-modulated light intensity approach for determination of optical properties in a two-layer medium, Weber et al. proposed a stepwise algorithm for sequential determination of the five free-parameters in a two-layer model from experimental diffuse reflectance arising from different spatial frequencies [43]. While allowing *l* as a fit parameter, the accurate recovery of bottom layer reduced scattering appeared impossible for any spatial frequency, and the fitting of other optical coefficients possessed extremely large errors depending on the spatial frequency at which diffuse reflectance was measured. Thus, a sequential fitting algorithm was proposed for the reasonably accurate fitting of some optical parameters under the condition of known and constant *l* within 25% of its true value. Fit results were analyzed in different cases in which subsets of the four optical properties were held constant, with the remaining left as free fit parameters. The authors suggest the use of ultrasound to recover *l* in the case of an *in vivo* implementation of this algorithm. Fawzi et al., in an inverse method to determine the four optical coefficients in a two-layered model from SRR curves, obtained fittings with low errors relative to known values but required *l* > 5 mm [44]. Wang et al. presented a method for determination of optical coefficients at three wavelengths in two-layered media using NNs given the input reflectance at five SDSs under the condition of known *l* [45]. In comparison to the known values of optical properties of each layer, moderate errors were observed which appeared to exhibit a dependence on layer thickness and orientation. In a subsequent work, the group introduced a modified algorithm in which two NNs were applied successively, reducing errors in fitted coefficients.

### 1.3. Motivations of this work

The choice between single- and multi-layer reference models for the inverse determination of optical properties of turbid media depends on one’s unique measurement case. In the context of accurate optical characterization, one must consider the loss in stability of fitted coefficients when applying a multi-layered model compared to those incurred in neglecting the layered nature of the medium in question. For applications such as near-infrared spectroscopy (NIRS) of the human head for monitoring brain hemodynamics, there exists evidence that a layered-model may be better suited than a single-layer model due to increased accuracy in fitted *µ_a_* [35]. Conversely, in applications in which one wishes to monitor the relative change of an optical coefficient in response to a stimulus, the increased stability of fitting provided by the single-layer model may be preferred over a more physically-accurate layered model [46]. Additionally, there exist scenarios in which existing multi-layered inverse algorithms may be unable to determine accurate fittings due to the necessity of significant *a priori* information. Most algorithms presented in literature require a well-known *l* to obtain accurately fitted layer-dependent *µ_a_* and *µ_s_*^’^, requiring a secondary measurement of layer thickness(es) by ultrasound or OCT [8,43] and a significant increase in measurement time. In the case of imaging SRR techniques, these extra measurements may also prevent non-contact functionality, a desirable quality of SRR imaging methods. Further, some multi-layer inverse algorithms require at least one of four (for a two-layer model) optical coefficients to be known prior to the inverse search – a potentially unsatisfiable condition in some applications. The argument presented in this work is that despite the increased theoretical or physical validity that layered models may provide, the fitting of optical properties obtained with such models currently possesses limitations in accuracy, stability, and practicality that may encourage researchers to instead use the simpler, more readily available single-layer simplifying model [16]. Thus, one must consider the errors that arise in making this often-necessary assumption.

While *µ_a_-µ_s_*^’^ cross-talk has been documented since about 30 years, there still exists very little analysis and characterization of the issue in physiologically-relevant scenarios. It is possible that there are many researchers that are not aware of the risk this phenomenon presents when making the single-layer assumption in SRR or similar studies, thus there exists a need for renewed attention for increased awareness and improved understanding of this phenomenon. Previous investigations of cross-talk in the context of diffuse reflectance computational and experimental studies have used only the diffusion approximation of the RTE and/or consider only a small spectral region [34,45,47,48]. To the knowledge of the authors, there exists no example in the literature of an analysis of the optical and geometrical regimes in which the single-layer model assumption results in elevated *µ_a_-µ_s_*^’^ cross-talk and/or systematic fitting errors that utilizes RTE analytical solutions, nor one that considers optical properties ranging many orders of magnitude. In this work, we construct two-layer volumes with physiologically-relevant *ex vivo* porcine tissue optical coefficients [49] to obtain spatially-resolved reflectance curves that are subsequently fitted by a single-layer model. A quantification of the risk due to *µ_a_-µ_s_*^’^ cross-talk according to the volume’s optical properties and geometry will serve to inform researchers in their choice of reference model in future SRR studies and their assessment of published optical properties for their applications.

## 2. Materials and methods

Analytical solutions to the RTE have been a focus of our group for over a decade, with several advances having been made, including obtaining solutions for cases of Fresnel-defined boundary conditions in semi-infinite regimes [50], single-scattered radiance [13], and for turbid layered media [51]. We describe here briefly the analytical solution to the RTE obtained for spatially-resolved reflectance in multiple-layered media that will be used to obtain results in this work, with details and validation presented elsewhere [51]. The time-independent RTE in a three-dimensional volume is given by Eq. (1), including a source with lateral beam profile described by *S*(*ρ*), with *ρ* a radial distance confined to the surface of the sample volume:

(1) $$\hat{s}\cdot \nabla I(r,\;\hat{s})+\mu_{t}I(r,\;\hat{s})=\mu_{s}\int I(r,{\hat{s}}^{\prime })f(\hat{s}\cdot {\hat{s}}^{\prime }){\text{d}}^{2}s^{\prime }+S(\rho )\delta (z)\delta (\hat{s}-\hat{z}).$$

**Fig. 1.**
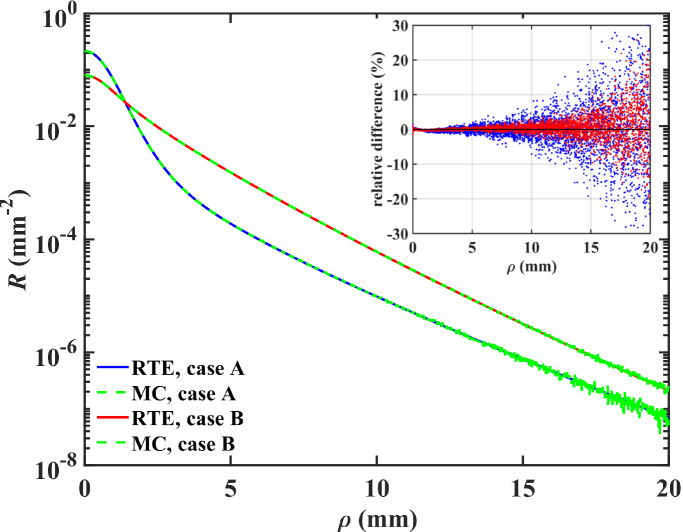
Comparison and relative difference between SRR curves obtained by analytical solutions of the RTE [51] and MC in a two-layer medium. Case A is characterized by *µ_a_*_,1_ = 0.005, *µ_s_*_,1_^’^ = 5.0, *µ_a_*_,2_ = 0.050, and *µ_s_*_,2_^’^ = 1.0 mm^−1^ respectively, with subscripts denoting the layers of the two-layer model. Both layers have *g* = 0.9, assuming a Henyey-Greenstein scattering phase function. Case B is characterized by *µ_a_*_,1_ = 0.001, *µ_s_*_,1_^’^ = 1.00, *µ_a_*_,2_ = 0.010, and *µ_s_*_,2_^’^ = 5.0 mm^−1^, and *g* = 0.5 for both layers. For both cases, *n* = 1.4, and reflectance collected with radial step size *dρ* = 0.1 mm. Curves arising from MC model are normalized to the incident 5 × 10^8^ photons.

The unit vector $\hat{s}$ is the propagation direction of a photon within the sample volume, with *I* representing the internal light radiance at a position **r**. The total attenuation coefficient *µ_t_* is related to absorption and scatter by *µ_t_* = *µ_a_* + *µ_s_*, and the scattering phase function is given by $f(\hat{s}\cdot {\hat{s}}^{\prime })$ , with $\int f(\hat{s}\cdot {\hat{s}}^{\prime }){\text{d}}^{2}s^{\prime }=1$ . In this work, we used a Gaussian beam profile, such that $S(\rho )=2/(\pi \;\rho_{\text{w}}^{2})\text{exp}\left(-2\frac{\rho^{2}}{\rho_{\text{w}}^{2}}\right)$ , with *ρ*_w_ the radius of the incident beam. The Kronecker *δ* function along with the depth *z* in the plane perpendicular to the volume surface denote that the beam profile is confined to the sample surface. The RTE is solved in the fashion of [52], in which an eigenvalue problem is formed once the two-dimensional Fourier transform and spherical harmonics, by the *P_N_* approximation [53], are invoked regarding the lateral and angular coordinates, respectively. Here, due to rotational symmetry (assuming a perpendicular incident light beam), the Fourier transform may be replaced by a one-dimensional Hankel transform. Thus, the solution is determined in the spatial frequency domain and numerically inverted to obtain the steady-state spatially-resolved domain solution, describing the *ρ*-resolved intensity acquired at the surface of the sample, that is used for all calculations in this work. Below, Fig. 1 demonstrates the agreement of reflectance *R* arising from our RTE solutions and those from simulations arising from an in-house developed MC code modeling the volume as a rectangular layer of finite-thickness stacked on top of a deeper semi-infinite layer. To obtain the results in Fig. 1, 5 × 10^8^ photons were simulated.

In this work, we calculated reflectance arising from analytical solutions to the RTE in semi-infinite geometries of either one or two unique layers. In each model, the origin in three dimensions was defined as the incident point of radiance on the sample, with virtual detectors confined to the sample surface and having positions defined by a radial SDS from the origin. For all simulations, *ρ*_w_ was set to 1 mm. Additionally, we invoked the rotationally-symmetric Henyey-Greenstein function to replace the scattering phase function in Eq. (1) and used *g* as an input parameter to obtain forward reflectance curves, working with the reduced scattering coefficient *µ_s_*^’^ where *µ_s_*^’^ = *µ_s_*(1-*g*). For all two-layer volumes considered in the remainder of this work, *n* and *g* were held constant across both layers at 1.4 and 0.9 respectively. The thickness of the superficial layer *l* was set to 1 mm and virtual detectors were positioned from 0 to 20 mm from the source with a radial bin width *dρ* of 0.1 mm. The forward reflectance curves were then fit with reflectance arising from a single-layer model using an LM optimization algorithm to determine effective *µ_a_* and *µ_s_*^’^ at each individual wavelength. The LM algorithm was used to optimize the cost function *χ*^2^, defined as

(2) $$\chi^{2}=\overset{N}{\underset{n=1}{\sum }}{\left[\frac{R_{s}(\rho_{n})-R_{t}(\rho_{n})}{\sigma (\rho_{n})}\right]}^{2},$$

where *R_s_* and *R_t_* are the reflectance arising from the single- and two-layer models respectively, and *σ* is the weighting function resulting in preferential weight given to *R* at certain *ρ*. Fittings obtained with *χ*^2^ are compared to those obtained with *χ*_log_^2^,

(3) $$\chi_{\text{log}}^{2}=\overset{N}{\underset{n=1}{\sum }}{\left[\text{log}(R_{s}(\rho_{n}))-\text{log}(R_{t}(\rho_{n}))\right]}^{2},$$

a common cost function in SRR studies chosen to increase the weight of reflectance experiencing larger penetration depth. To ensure a reasonable fitting procedure similar to one which may be used in an experimental measurement, only three orders of magnitude of reflectance contribute in the inverse fitting algorithm are considered. The reflectance contributing to the fitting are those between the limits of *R_t_*(*ρ*_1_) and *R_t_*(*ρ_N_*), where *ρ*_1_ is the largest of either *ρ*_w_ + *dρ* or 1/*µ_s_*_,1_^’^, and *ρ_N_* is the radial distance at which *R_t_*(*ρ*_1_)/*R_t_*(*ρ_N_*) = 10^3^. In presented fittings in the following sections, the choice of *σ* in the calculation of *χ*^2^ is specified as one of two approaches, unity weighting with no dependence on *ρ*, *σ* = 1, and $\sigma =\sqrt{R_{t}(\rho )}$ , noting that their units are not rigorously treated here. The choice of $\sigma =\sqrt{R_{t}(\rho )}$ is used as an example to demonstrate fittings in which *R* at large *ρ* are weighting more heavily than with the use of *σ* = 1 but not to such an extent as with a *χ*_log_^2^ cost function.

## 3. Results

### 3.1. µ_a_-µ_s_^’^ cross-talk

**Fig. 2.**
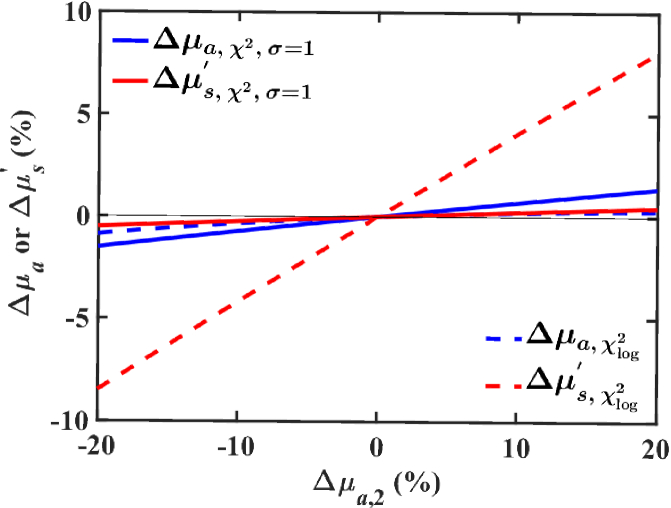
Definition of *µ_a_-µ_s_^’^* cross-talk demonstrated in a two-layer model. The change in fitted single-layer coefficients Δ*µ_a_* and Δ*µ_s_^’^* is plotted against Δ*µ_a,_*_2_, the relative difference in *µ_a_*_,2_ compared to its baseline at 0% perturbation. Absolute values of baseline optical properties are *µ_a_*_,1_ = 0.073, *µ_s_*_,1_^’^ = 4.05, *µ_a_*_,2_ = 0.020, and *µ_s_*_,2_^’^ = 2.18 mm^−1^, taken from *ex-vivo* porcine measurements of dermis (1) and adipose (2) tissues at 500 nm [49].

The phenomenon of *µ_a_*-*µ_s_*^’^ cross-talk is demonstrated by an example in which single-layer effective optical coefficients are fitted in response to small perturbations in only one optical coefficient within the two-layer model, where the subscripts 1 and 2 are used to denote the outer and deeper layers respectively. Compared to a baseline, *µ*_*a*,2_ was perturbed by -20 to 20% in 0.4% steps, while *µ*_*a*,1_, *µ*_*s*,1_^’^, and *µ*_*s*,2_^’^ were held constant. The percent difference of effective *µ_a_* and *µ_s_*^’^ at each perturbation relative to those fitted at baseline coefficients are given by Δ*µ_a_* and Δ*µ_s_*^’^ and shown in Fig. 2.

The *µ_a_-µ_s_*^’^ cross-talk is evident in fittings obtained with both cost functions; however, the effect is most extreme in the case of *χ*_log_^2^ fittings, in which light of deeper penetration depth contributes most heavily in the calculation of the cost function. Despite perturbations having been applied only to the absorption in the deeper layer, sympathetic deviation in Δ*µ_s_*^’^ away from 0% is present in fittings of single-layer optical coefficients. Further, this compensatory increase in the magnitude of Δ*µ_s_*^’^ in response to a change only in deeper layer absorption is many times larger than Δ*µ_a_*, which is unexpected. Thus, the definition of *µ_a_-µ_s_*^’^ cross-talk is demonstrated by Fig. 2 as the compensatory change in one optical parameter (of either *µ_a_* or *µ_s_*^’^) in response to a change in the other.

### 3.2. Spectrally-resolved fittings of µ_a_-µ_s_^’^

**Fig. 3.**
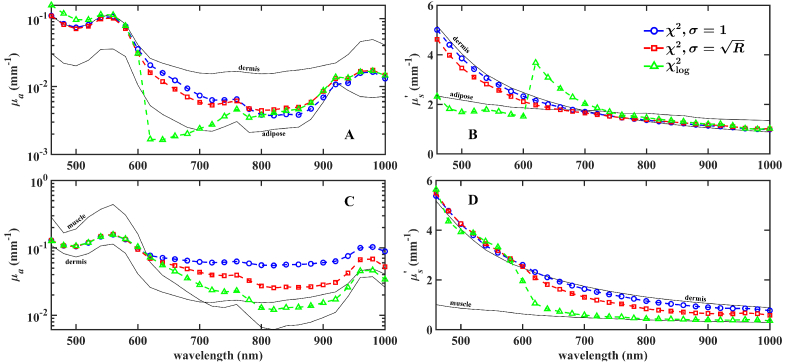
Spectrally-resolved fittings of two-layer reflectance curves obtained from RTE solutions arising from two porcine models. Top left (A) and right (B) results obtained from dermis-adipose model and bottom two plots (C and D) arise from dermis-muscle model. For each, three combinations of cost function and weightings are used to obtain single-layer effective fittings of *µ_a_* and *µ_s_*^’^. Solid lines show the *ex vivo* measured values of absorption and reduced scatter as a reference for the viewer, taken from [49]. Dashed lines are a guide for the eyes.

To demonstrate the practical consequences of *µ_a_-µ_s_^’^* cross-talk in experimental SRR measurements of layered media optical properties, a two-layer volume is constructed from recent porcine *ex-vivo* optical properties measured by integrating sphere [49]. Two-models are compared: porcine dermis-adipose and dermis-muscle models, in which dermis is always the superficial layer, with the procedure stated in Section 2 applied to obtain spectrally-resolved fittings in the wavelength range of 460 to 1000 nm in 20 nm steps. Forward reflectance was fitted to obtain effective absorption and reduced scatter in a single-layer model. Fittings were obtained with three combinations of cost function and weighting method: *χ*^2^ and *σ* = 1, *χ*^2^ and $\sigma =\sqrt{R_{t}(\rho )}$ , and *χ*_log_^2^ and shown in Fig. 3.

Fittings of optical coefficients in the porcine models demonstrate multiple risks that arise when working under the single-layer assumption. Figure 3(A and B) shows that fittings obtained with the dermis-adipose model under both weighting methods of the *χ*^2^ cost function appear reasonable, with fittings appearing to be some kind of weighted average of those of the individual layers. We see an expected result at low wavelengths, where the superficial layer absorption and reduced scattering are large, single-layer fittings adhere closely to that of the dermis due to the low light penetration. Fittings obtained with a *χ*_log_^2^ cost function, however, feature unexpected fittings, with *µ_a_* and *µ_s_*^’^ exhibiting spectral regions in which fittings are smaller than those of either individual layer in the two-layer model. Further, both fitted coefficients display spectral discontinuity with clear deviations from those displayed by the coefficients of individual layers. Figure 3(C) shows a fitted absorption arising from the dermis-muscle model with the *χ*^2^ cost function that is reasonable at wavelengths less than 600 nm, with values falling within those of individual layers and slightly favoring that of the superficial dermis layer. At longer wavelengths, however, fitted absorption remains nearly an order of magnitude larger than those of the individual layers in the case *σ* = 1 and changes significantly depending on the choice of cost function and weighting combination. Similar to the behavior observed in the dermis-adipose model, *µ_s_^’^* fittings in Fig. 3(D) exhibit regions of smooth spectral trends in both weighting cases of *χ*^2^ and discontinuity in those obtained with *χ*_log_^2^. We observe a unique behavior in the dermis-muscle model fittings obtained with *χ*^2^: an overestimation of *µ_s_*^’^ and simultaneously reasonable fittings in *µ_a_* at wavelengths of 600 nm and shorter.

### 3.3. µ_a_-µ_s_^’^ search space

**Fig. 4.**
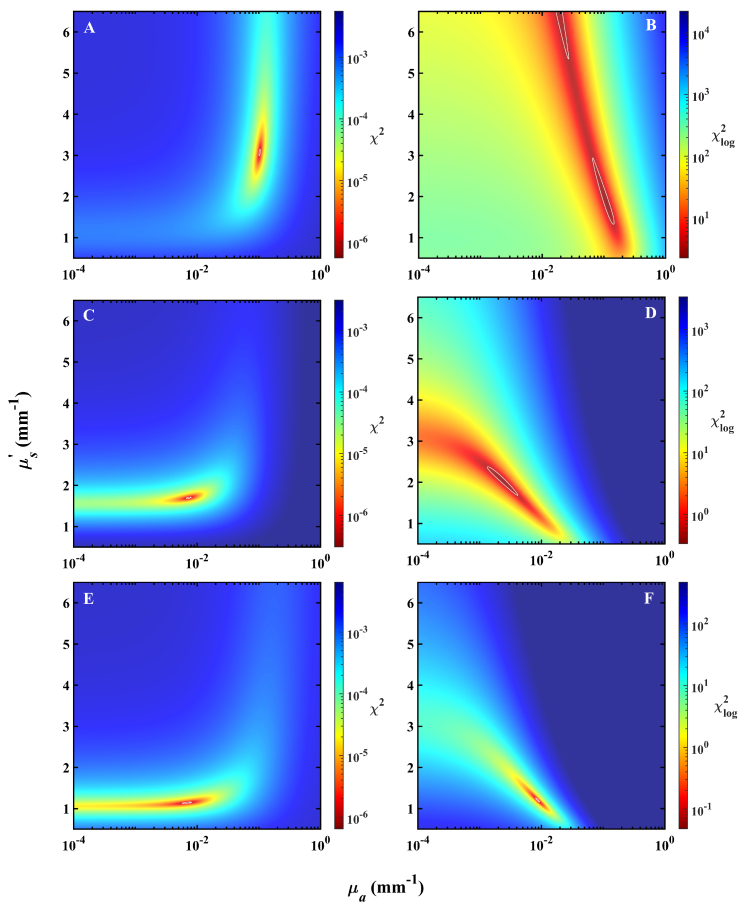
Two-dimensional *µ_a_*-*µ_s_*^’^ heatmaps showing the LM search space for the porcine dermis-adipose model. Plots are shown for 540 (A and B), 700 (C and D), and 900 (E and F) nm from top to bottom row respectively. White contour lines denote cost that is 50% larger than the minimum in the entire search space to serve as a reference to the viewer.

**Fig. 5.**
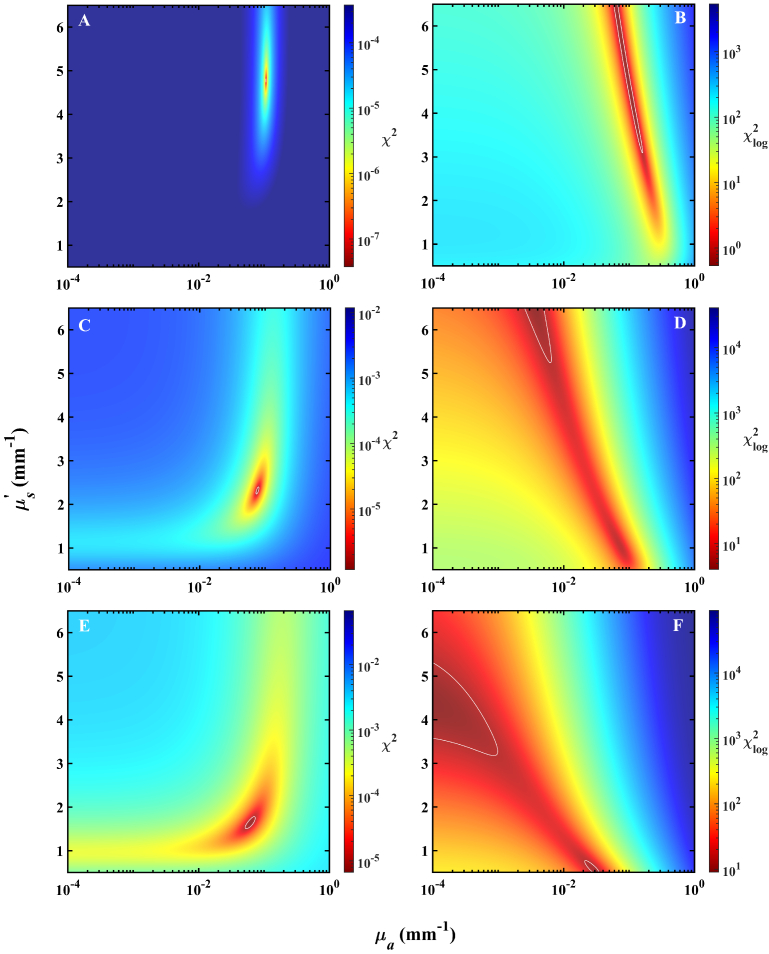
Two-dimensional *µ_a_*-*µ_s_*^’^ heatmaps showing the LM search space for the porcine dermis-muscle model. Plots are shown for 480 (A and B), 620 (C and D), and 700 (E and F) nm from top to bottom row respectively. White contour lines denote cost that is 50% larger than the minimum in the entire search space to serve as a reference to the viewer.

To explore the behavior shown in Fig. 3, the two-dimensional *µ_a_*-*µ_s_*^’^ search spaces over which the LM optimization is performed for each model are shown in Figs. 4 and 5. A 300 by 300 grid of unique combinations of *µ_a_* and *µ_s_*^’^ over physiologically relevant regions was constructed, with single-layer forward reflectance calculated at each pixel. *χ*^2^ and *χ*_log_^2^ relative to two-layer reflectance arising from the models described in Section 3.2 at three wavelengths were calculated and plotted with a colormap applied to show regions of equal cost in *µ_a_*-*µ_s_*^’^ space. Shown here with the *χ*^2^ cost function is only the case of *σ* = 1.

LM search spaces obtained with *χ*^2^ are clearly single-valued with very small regions of optimal cost function. In this case, LM-determined fitted optical coefficients have excellent repeatability and exhibit no dependence on initial guess in the two-dimensional space, with guesses in *µ_a_* differing even by orders of magnitude from the best position having no effect on the LM algorithm’s ability to find the global minimum. Fittings obtained with *χ*_log_^2^, however, display a stretching of the optimal region and the appearance of clearly demarcated local minima. In the case of 700 nm, when local minima are not present, there still exists a significantly larger area of low *χ*_log_^2^ compared to *χ*^2^ at the same wavelength, suggesting the possibility of decreased precision in fitted coefficients for the former cost function. Depending on the wavelength, it appears that there may or may not exist local minima in the *χ*_log_^2^ optimization space.

The LM search spaces of the dermis-muscle and dermis-adipose models appear similar. Again, the *χ*^2^ distributions feature very sharp minima and never exhibit local minima. The stretching of optimal *χ*_log_^2^, however, appears more detrimental in this model than the previous, with regions of low cost spanning large regions of optical coefficients that appear, in comparison to those of individual layers, quite far from physically-reasonable values. The minimum *µ_a_* accessible in the LM search space was 2 × 10^−6 ^mm^−1^, and it appears that the *µ_a_* coinciding with the minimum *χ*_log_^2^ may reside outside the lower bound of the search range. The discontinuities in spectral fittings of the dermis-muscle model of Fig. 3(C and D) are explained by its *µ_a_*-*µ_s_*^’^ search space at 620 nm which displays lengthening of low *χ*_log_^2^ regions and formation of local minima, resulting in the LM-determined optimal combination of *µ_a_* and *µ_s_*^’^ not matching with those at the true global minimum *χ*_log_^2^. Further, it appears that for multiple wavelengths and in both models, even if the LM algorithm were able to find the true global minimum *χ*_log_^2^, the fitted combination of optical coefficients would not be physically reasonable.

### 3.4. Superficial layer thickness

Up to now, all results presented were obtained with an *l* = 1.0 mm. We show here a case-study demonstrating the effect of superficial layer thickness on fitted single-layer effective optical coefficients. The porcine dermis-muscle model introduced in Section 3.2 is used, with optical properties taken at two wavelengths: 600 and 800 nm. At each, *l* is varied from 0 to 7 mm in 0.035 mm steps, and two-layer forward reflectance is calculated and subsequently fitted by the single-layer model with the same three combinations of cost function and weighting presented in Section 3.2.

**Fig. 6.**
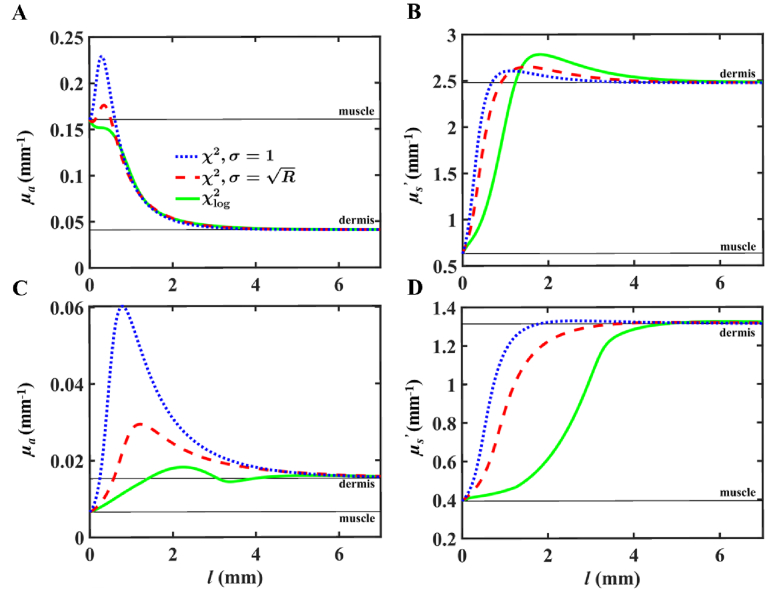
Single-layer optical coefficient fittings arising from a two-layer dermis-muscle volume with varying superficial layer thickness *l* and two wavelengths of 600 (A and B), and 800 nm (C and D). Solid black lines denote the reference values for each coefficient of each tissue type. Absolute optical properties of the two-layer media at 600 nm are *µ_a_*_,1_ = 0.041, *µ_s_*_,1_^’^ = 2.48, *µ_a_*_,2_ = 0.161, and *µ_s_*_,2_^’^ = 0.632 mm^−1^, with 1 and 2 denoting dermis and muscle respectively. At 800 nm, *µ_a_*_,1_ = 0.015, *µ_s_*_,1_^’^ = 1.31, *µ_a_*_,2_ = 0.007, and *µ_s_*_,2_^’^ = 0.394 mm^−1^.

Figure 6 shows that when *l* = 0 mm and the two-layer volume collapses to a single-layer, fitted coefficients match those of the deeper muscle layer. Further, when *l* is large, the two-volume can be treated effectively as a single-layer, with fitted optical coefficients converging to those of the superficial dermis layer. At these extreme cases of *l*, fitted *µ_a_* and *µ_s_*^’^ are independent of cost function and weighting method, a validating result that is expected. Between these bounds, however, fitted coefficients exhibit a remarkable behavior that is attributed to the disagreement between reflectance curves arising from the single- and two-layer models. At low *l*, we observe a similar behavior as that which was seen in Fig. 3: overestimation of *µ_a_* in comparison to that of the individual tissue types. This overestimation is seen for both choices of *σ* during the use of *χ*^2^, however its magnitude is far greater in fittings obtained with *σ* = 1. The use of *χ*_log_^2^ reduced but did not eliminate overestimation of *µ_a_*. Further, oscillatory behavior in fitted absorption appears as a function of *l* when this cost function is used – a phenomenon not seen otherwise. The trends with *l* of fitted *µ_s_*^’^ are not significantly different in form throughout the different choices of cost and weighting method, and there is an overestimation for each in comparison to the *µ_s_*^’^ of individual tissue types, though we note that the degree of overestimation is far larger in *µ_a_* than in *µ_s_*^’^.

## 4. Discussion

We presented in Fig. 2 a generic case of absorption-scatter crosstalk in a simulated porcine model constructed with *ex vivo* integrating sphere measurements. We demonstrated that, depending on the choice of cost function and weighting method, significant compensatory response in the opposite optical parameter may be present when one is finely adjusted, suggesting that there exist regimes in which errors arising from the single-layer assumption far outweigh the sensitivity of measurement of small variations in absorption. This transfer of information regarding the measurement volume from one optical coefficient to another must be considered during functional measurements of a single optical coefficient in response to a physiological stimulus.

Using two case studies of porcine dermis-adipose and dermis-muscle models, we presented in Fig. 3 spectrally-resolved absorption and reduced scattering coefficient fittings using a single-layer reference model. Unique effects of the single-layer assumption are seen in the results of each model, with systematic over- and underestimation of fitted parameters and discontinuity between fittings of different wavelengths within the same model. The dermis-muscle model features overestimation of fitted absorption in the case of deep layer *µ_s_*^’^ much smaller than that of the superficial layer. One would expect the single-layer fitted effective optical coefficient to be some kind of average of the those of each layer in a multi-layered volume, weighted by *l*, SDS, beam diameter, etc. The findings demonstrated in the spectral fittings suggest that under some conditions, there is a fitted absorption that is larger than that of either region in the two-layer forward model. In the results of a previous work, in which similar fittings were performed under the constraint of equal absorption across both layers, the degree of overestimation was seen to be sensitive to the ratio of reduced scattering in the two-layer model [40]. The same behavior can be seen in this generic case, in which the absorption across the two layers is not equal. In this example, the fittings obtained in the dermis-adipose model appear reasonable, as the only criterion used to assess “reasonability” is that the fitting falls within those of the individual layers. It is likely that, despite possessing a reasonable value, fitted *µ_a_* may still possess an influence from the single-layer assumption that is not detectable. In a practical measurement without the *a priori* knowledge of the true coefficients of each layer, one cannot determine whether such errors are present and must assume the risk. We note that all fittings obtained during this work are absolute fittings between the two- and single-layer reflectance curves. For a consideration of optical properties obtained by a relative fitting procedure using a third free parameter participating in the LM fitting, one is directed to our recent work [40]. We also note that in fittings not shown, similar results as those presented here have also been obtained using a smaller beam radius of *ρ*_w_ = 0.2 mm.

A unique case of Fig. 3 is considered here, in which both porcine models near 700 nm possess reduced scatter that is roughly equal across the two-layers of the dermis-adipose model. Also at this wavelength, the absorption is approximately equal across the two-layers in the dermis-muscle model. Under the condition of equal reduced scatter across both layers, fittings with of the dermis-adipose model display a *µ_s_^’^* obtained with *χ*^2^ that is roughly equal to those of the individual layers and a reasonable *µ_a_*. The use of *χ*_log_^2^, however, results in a fitted *µ_s_^’^* that is larger than expected and *µ_a_* outside the range of the individual layers. The dermis-muscle model fitting, in the case of roughly equal absorption, displays reasonable fittings of *µ_s_^’^* but overestimation of *µ_a_* for all combinations of cost and weighting functions. These findings suggest that the partial simplification of a two-layer media into one in which the two-layers are identical in either optical coefficient does not appear to be enough to eliminate the effect that arises due to disagreement between reflectance arising from the two models. It may be useful to assess whether a multiple-layer inverse algorithm is able to determine accurate fitted coefficients under such partially-simplifying conditions.

We then investigated the spectral discontinuity of fitted optical coefficients by analyzing the two-dimensional LM algorithm search space. Prior results of Figs. 2 and 3 demonstrate that fittings are extremely sensitive to the choice of cost function. In Figs. 4 and 5, we observed a significantly different cost function distribution between the choices of *χ*^2^ and *χ*_log_^2^, with positions of minima in the two cases differing at times by many orders of magnitude in *µ_a_*. There was significant stretching of regions of low *χ*_log_^2^ compared to clearly single-valued spaces of *χ*^2^, however the minimum obtained with *χ*^2^ was often located at a physically unreasonable location. Thus, even if we were to use an optimization approach which is more robust to the presence of local minima in the search space, such as a LUT or particle-swarm-optimization [54,55], the recovered optical properties may still be distant from those of either of the two layers or non-physical. The LM-fitted optical coefficients were consistent across multiple searches in the case of *χ*^2^, however for *χ*_log_^2^, the fitting result displayed dependence on the starting position in the search space. We demonstrate with two case studies the high degree of variation that an inverse algorithm search space may exhibit, suggesting that in a similar diffuse reflectance study of multiple-layer media, a researcher should take care in considering that the geometric and spectrally-dependent optical properties of their unique model may strongly influence their two-dimensional *µ_a_*-*µ_s_^’^* search space. Further, a researcher may consider the choice of inverse algorithm to best navigate spaces that are not well-posed.

Finally, we considered in Fig. 6 the fittings of optical coefficients in response to superficial layer thickness in a two-layer model. The aforementioned phenomenon of absorption coefficient overestimation was clearly shown along with the effect of cost function and weighting. The choice of *χ*_log_^2^ results in a lesser degree of overestimation of *µ_a_* than either choice of weighting with *χ*^2^, but displays the opposite trend in fits of *µ_s_*^’^. When using a cost function of *χ*^2^, the overestimation of coefficients as functions of *l* is less pronounced when $\sigma =\sqrt{R_{t}(\rho )}$ is used in comparison to *σ* = 1. This sensitivity to weighting demonstrates further that the reflectance at low SDS and large SDS do not agree for the same optical coefficients. It is notable to mention that the overestimation of absorption is largest for *l* in the range of about 0.25 to 1 mm, with a dependence of the peak position on the unique combination of the four optical coefficients of the two-layer forward model. It appears that the choices of cost function and weighting in the LM search are highly influential to both the absolute value of fitted coefficients and their trend with *l.* We show clearly that the depth sensitivity that increases with *χ*^2^, $\sigma =\sqrt{R_{t}(\rho )}$ and *χ*_log_^2^ is in direct response to the single-layer assumption of a two-layer medium. This result may provide researchers with an estimation of the errors in both absorption and reduced scatter that one may expect according to their unique model’s outer layer thickness.

## 5. Conclusion

The major findings of this work contribute to the conclusion that there exists a fundamental incompatibility between the reflectance from two-layer and single-layer volumes. Results obtained here describing the formation of local minima in the LM search space suggest that multiple combinations of optical properties in a single-layer model characterize a two-layer reflectance curve equally well. The disagreement in fittings obtained with different cost function and weighting combinations clearly demonstrates that identical optical coefficients cannot simultaneously describe light of significantly different penetration depth in the same volume. We have provided in this work a thorough description of the errors that one may expect when approximating a two-layer volume as a single-layer during diffuse spectroscopic experimentation. Considering the current limitations of multiple-layer inverse models in fitting accuracy and the burden of required *a priori* information, the choice of a single-layer volume remains very common, but as demonstrated in this work, one must be aware of the risks that such a simplifying assumption poses to the accuracy of *in vivo* determined optical properties by spatially-resolved reflectance methods.

## Data Availability

Data underlying the results presented in this paper are not publicly available at this time but may be obtained from the authors upon reasonable request.
